# Revisiting the overlooked role of recycled sewage water in high-income countries in adenoviral outbreaks such as the “2022 pediatric hepatitis’ outbreak”

**DOI:** 10.1186/s43054-022-00113-2

**Published:** 2022-08-25

**Authors:** Antoine AbdelMassih, Aya Kamel, Ali Mohamed Zaki, Ayten Aboudeif, Clara Emad, Dina Ramadan, Hanya Gaber, Harvey Bastorous, Mehraiel Shaker, Nancy Salah, Nourhan Hany, Nur El-Mestkawy, Rana Adel Naguib Sawiris, Rana Mamdouh, Sandy Atalla, Sara Abozeid, Sarah Ismail Ghazi, Steven A. Youssef, Youssef ElMaghraby, Zainab Khudhair, Rafeef Hozaien, Nadine El Husseiny, Meryam El Shershaby

**Affiliations:** 1 Pediatric Department, Pediatric Cardiology Unit, Cairo University Children Hospital, Faculty of Medicine, Cairo University, Kasr Al Ainy Street, Cairo, 12411 Egypt; 2 Cardiac Sciences’ department, Pediatric Cardiology Division, Sheikh Khalifa Medical City, Abu Dhabi, UAE; 3 Student and Internship research program (Research Accessibility Team), Faculty of Medicine, Cairo University, Cairo, Egypt; 4 Student and Internship research program (Research Accessibility Team), Faculty of Medicine, New Giza University, Giza, Egypt; 5 Faculty of Dentistry, Cairo University, Cairo, Egypt; 6 Pixagon Graphic Design Agency, Cairo, Egypt

**Keywords:** Adenovirus 41, Recycled sewage water, Unknown hepatitis cases, COVID-19

## Abstract

**Background:**

On the 5th of April 2022, cases of adenovirus-induced hepatitis were reported in Scotland and then reached multiple parts of the world. While adenovirus normally presents with diarrhea, vomiting, and fever, these novel cases also resulted in the development of fulminant hepatitis in non-immunocompromised cases.

**Main body:**

The responsible pathogen “Adenovirus 41” is an enterovirus. Enteroviruses are spread by the fecal-oral route and are resistant to drying. As such, they predominate in sewage water. Hepatitis is normally restricted to poorer countries, yet this new wave seems to be confined to mostly high-income countries in Europe and the USA. These countries treat and recycle a higher percentage of sewage water. We also propose that the fulminant nature of this strain could be due to either a cross-species mutation or the general decrease in trained immunity post-COVID-19 lockdown.

**Short conclusion:**

Evidence strongly suggests that the link between these new hepatitis cases is recycled sewage water. This should warrant further investigations on the origin of this outbreak by re-visiting the role of recycled sewage water in causing such outbreak.

## Background

A global surge of inexplicable hepatitis cases came into light on April 5, 2022, with the first cases reported in Scotland (1). The number of cases has since progressively risen all over the world. It is attributed to enteric adenovirus type 41 and presented with diarrhea, vomiting, and fever in children. It had not previously led to the development of fulminant hepatitis in non-immunocompromised cases [1].

To date, twelve European countries have reported cases. Intriguingly, the countries belong predominantly to the high-income category (according to the World bank). Fifty-five cases have been recorded from around the European Union/European Economic Area (EU/EE) as of April 27, 2022. The countries in question are Austria, Belgium, Denmark, France, Germany, Italy, Ireland, Norway, Poland, Romania, Spain, and the Netherlands. The cases started showing symptoms in early to mid-March and some tested positive for adenovirus [2].

### UK

Since the last report on April 25, active case-finding investigations have uncovered 34 confirmed cases, bringing the overall number of cases to 145, 128 of which originated in England. Fortunately, no deaths have occurred. (2) Four cases were reported in Ireland in children between the ages of 2 and 11. (3) In Scotland, 13 cases were confirmed, with a median age of 3.9. All 13 children were hospitalized at the time of publishing, and three of them required liver transplant assessment. One of the children received a successful transplant, while five of the thirteen patients are being treated in the hospital [3].

### Denmark

Denmark reported five instances in children under the age of ten and one in children above ten. There have been no deaths or need for liver transplants in the cases [2].

### Italy

Italy had 17 cases across various Italian regions. They suspected eight cases and nine are yet to be classified. The ages of the cases have not been confirmed, but all children are under 16 years of age. One case required a liver transplant [2].

### France

France reported two cases in children younger than 10 years old. Both cases had severe acute hepatitis, but neither required liver transplantation [2].

### Israel

Only 12 cases have been reported and are all hospitalized, and no further information is available [4].

### Spain

Not much information is available about the cases in Spain. The last report by the CDC on 23 April 2022 stated that there were 13 cases in Spain. The need for a hepatic transplant, mortalities, and hospitalization information has not been revealed [2].

### USA

Among 24 states, 109 cases have been reported in the USA, 14% need liver transplants, and nine require hospitalization. Five deaths were reported, but the states have not yet been revealed. The cases were reported in Alabama, Arizona, California, Colorado, Delaware, Florida, Georgia, Idaho, Illinois, Indiana, Louisiana, Michigan, Minnesota, Missouri, North Carolina, North Dakota, Nebraska, New York, Ohio, Pennsylvania, Tennessee, Texas, Washington, and Wisconsin. Alabama declared the most cases among the US states, 9; all of whom tested positive for adenovirus [5].

Table 1 shows the details of cases and their need for liver transplantation.

**Table 1:** Overview of cases of the number of unknown hepatitis cases and their need for liver transplantation until the 27th of April 2022

Countries	Number of cases	Number of deaths	Number of patients requiring liver transplantation
Austria	2	0	0
Belgium	2	0	0
England	128	0	10
France	2	0	0
Germany	1	0	0
Ireland	4	0	0
Italy	17	0	1
Israel	12	0	0
Netherlands	4	0	3
Norway	2	0	0
Poland	1	0	0
Romania	1	0	0
Scotland	13	0	3
Spain	13	1	0
USA	109	5	15
Totals	311	6	32

*Abbreviations*: *USA* United States of America

As mentioned earlier, most of the cases have tested positive for adenovirus 41. Adenovirus 41 is known to grow in sewage water. High-income countries rely on recycled sewage water to fight water scarcity; therefore, we hypothesize that recycled sewage water is incriminated in this outbreak. We will also discuss the theories behind the fulminant nature of the observed hepatitis despite occurring in immunocompetent hosts.

## Main body

### Enteric adenoviruses, predominant in sewage water?

There are 47 serotypes of human adenoviruses (hADV) divided into six subgenera (A-F). The “F” subgenera include enteric adenoviruses type 40 and 41 that cause gastroenteritis. These types constitute the second most important cause of infantile gastroenteritis. They mostly affect children under two, causing diarrhea in 4–17%. Clinically, the infection appears as vomiting, watery diarrhea, mild dehydration, and low-grade fever; a specific symptom is a protracted diarrhea [6].

Adenoviruses enter the body through the mouth, the nasopharynx, or the conjunctiva. Because of the extended shedding of viruses in feces, fecal-oral transmission accounts for most adenovirus infections in young children. Adenovirus infections of the nose, throat, and eyes are linked to insufficiently treated swimming pool water. They survive longer in water and are resistant to drying; therefore, contaminated inanimate surfaces may play a substantial role in viral transmission [7].

The pathogenic viruses are discharged into the environment through treated and untreated wastes (sewage water may undergo three levels of treatment: primary, secondary, and tertiary) [8]. A study measured the adenovirus level in surface water, wastewater, and combined sewer overflows (CSOs) using PCR. Adenoviruses were consistently present in sewage and were occasionally detected in rivers receiving sewage effluents. Unfortunately, PCR cannot determine the viability of the virus. Virus detection is also not attempted until after the outbreak, inhibiting prophylactic measures that could decrease outbreak severity [9].

Adenoviruses are also resistant to deactivation via monochromatic UVC light and monochloramines. These treatments have minimal effects on the viral structural proteins because the infectivity of adenoviruses does not heavily depend on its DNA being fully intact—what the above treatments target. However, the virus can be inactivated by free chlorine and full-spectrum sunlight. The extent of inactivation of the human adenoviruses correlates to the damage to the protein structures of the virus as it seems to readily repair DNA damage caused by UVC, explaining the infectivity of said treatment [10].

### Sewage water re-use is a hallmark of high-income countries, is it a coincidence?

Western Europe reuses 16% of its treated wastewater, while MENA reuses 15%. Western Europe has the highest rate of treating wastewater (86%) compared to Saharan Africa and South Asia (16%). High-income countries treat 74% of their wastewater compared to 4% treatment in the low-income countries; wastewater treatment increases proportionately with a country’s income.

For instance, in Europe, 71% of industrial wastewater produced is treated, while in Latin America, only 20% undergoes treatment. In the Middle East and North Africa (MENA), 51% of industrial wastewater undergoes treatment. African countries lack financial resources for wastewater development which is a constraint on wastewater management. All 32 of the 48 countries in sub-Saharan Africa had no available data neither on wastewater production or treatment.

In six member countries (Germany, Latvia, Luxembourg, the Netherlands, Austria, and Sweden) and Switzerland, and the UK, the proportion of the population connected to secondary treatment plants has increased further than 95%. On the other side of the scale, less than one in two households are connected to at least secondary urban sewage treatment facilities in Cyprus, Malta, Romania, and Croatia (all 2018 data). Over the period shown, some countries have succeeded in significantly expanding the scope of wastewater treatment; for instance, Hungary (29.8 to 80.4%) and Slovenia (12.3 to 68.9%) [11].

Almost 90% of wastewater in Israel is treated, most of it in agricultural irrigation 18,000 WWTPs, and millions of kilometers of pipes. The majority of wastewater is reused in Israel, while Europe recycles merely 60%. They are aiming to recycle the entire volume in the future [12].

We can deduct from the above that it is considerable overlap between the distribution of cases of this new outbreak and the percentage of reliance on recycled or treated sewage water. This might signify that the use of recycled sewage water, as mentioned might be implicated in the observed outbreak [12].

### Why is the observed cases fulminant?

Enteric adenoviruses can induce a mild hepatitis illness. The occurrence of more fulminant forms of hepatitis is linked to a state of immunocompromise. According to the ongoing reports, affected patients were not suffering from any concurrent immunodeficiency or under treatment by immunosuppressive medications. The hygiene theory has gained popularity in justifying the fulminant nature of hepatitis observed in this outbreak [13, 14].

#### COVID-19 and the hygiene theory

The COVID-19 pandemic induced a worldwide lockdown to decelerate the spread of the SARS-CoV-2 virus. While this was crucial to limit the spread of SARS-COV2, it deleteriously altered the seasonal trends of viral infections. As a case in point, Maison et al. recorded an unusual surge of both Rota and Noroviruses post lockdown. Furthermore, during the lockdown, some non-respiratory infections increased as seen by the exacerbation of dengue reported in Singapore [15].

This could be justified by the hygiene hypothesis and the absence of trained immunity. The hygienic precautions implemented alongside the lockdown reduced exposure to pathogens diminishing the stimulation of the nonspecific immune system and altering the balance of commensal microorganisms [16]. The limitation of the functional interaction of the immune system is evident by the unexpected increase of RSV cases showing similar viral genotypes as those diagnosed in previous years post lockdown in Greece. Similar findings were found in Australia where the seasonal RSV peaks exceeded that of previous years [17, 18].

At the genetic level, this can be corroborated by studies showing that socially isolated mice and other species show a downregulation of anti-viral genes. Moreover, the sedentary lifestyle, confounded with the unhealthy diets attributed to the limited income found during the pandemic-induced economic crisis, led to a higher prevalence of obesity, which affects the immune system. Evidence suggests that this unhealthy lifestyle may have upset the microbiota and adversely impacted the production of antibodies already showing “inexperienced” responses to foreign antigens in children compared to adults [19].

#### The mutated strain theory

Another hypothesis for fulminant hepatitis occurring in this outbreak is that a mutated strain of ADV 41 could have acquired augmented virulence allowing it to induce more liver damage. Interestingly, we found two reports dating back to 2011 and 2013 describing similar illnesses in monkeys and marmosets by a mutated strain of ADV [20, 21].

A catastrophic outbreak of fulminant pneumonia and hepatitis was documented in a confined colony of New World titi monkeys of the *Callicebus* genus at the California National Primate Research Center (CNPRC). One third of the monkeys got infected, and 83% of them died. Virochip was used to identify a unique and highly divergent adenovirus as the source. Clinical and serological evidence further demonstrated that this virus infected a CNPRC researcher and a family member. Thus, researchers revealed the possibility of adenovirus cross-species infection [12]. Furthermore, SqMAdV-1, a novel adenovirus discovered in immunocompromised squirrel monkeys, has fatal effects in species aside from its natural host [20].

In 2011, an experiment was conducted to show cross-species TMAdV infection of marmosets. An acute mild respiratory disease, associated with a rise in virus-specific neutralizing antibody titers, was generated by the nasal administration of a cell cultured-adapted TMAdV strain into three marmosets [21].

Reports circulated of fulminant hepatitis in a Japanese aquarium afflicting otariids; presenting with symptoms of diarrhea which acutely progressed to low spirits and death after 3 days. Available data suggest that increased aspartate aminotransferase, alanine aminotransferase, necrotic hepatitis, and eosinophilic intranuclear inclusion bodies [22].

## Conclusion

The reported cases of adenovirus-induced hepatitis are mainly circulating in high-income countries. Given the pattern of survival of adenovirus in sewage water, its resistance to inactivation by ultraviolet rays and the increasing reliance of high-income countries on recycled sewage water for drinking water and for irrigation of crops, and it is advisable to test the possible incrimination of recycled sewage water in such outbreak. If the suggested hypothesis is proven true, recycling of sewage water should take into consideration resistant pathogens such as adenoviruses.

**Fig. 1:**
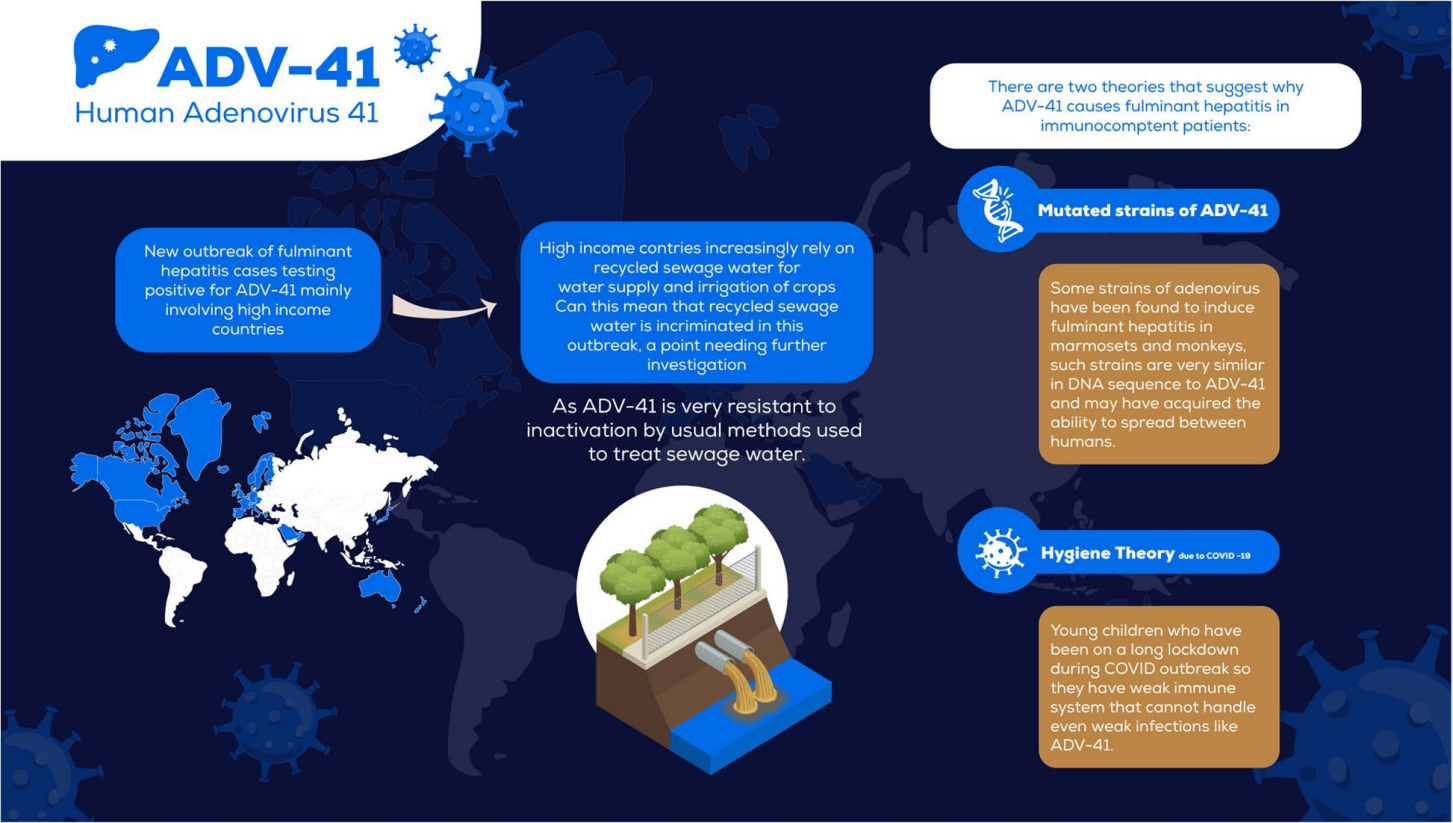
Can be sewage water recycling be implicated in the unknown hepatitis outbreak. Abbreviations: ADV41, adenovirus 41; COVID-19, coronavirus disease 2019

Figure 1 summarizes the points discussed in this article.

## Data Availability

Not applicable

## Abbreviations

ADV: Adenovirus
CDC: Center for disease control
COVID-19: Coronavirus disease 2019
CSO: Combined sewer overflows
DNA: Deoxyribonucleic acid
EU: European Union
PCR: Polymerase chain reaction
SARS-COV-2: Severe acute respiratory syndrome 2
SqADV: Squirrel adenovirus
TMADV: Titi-monkey adenovirus
UK: United Kingdom
USA: United States of America
UVC: Ultraviolet-C
